# Breeding for Resistance to Fusarium Wilt of Tomato: A Review

**DOI:** 10.3390/genes12111673

**Published:** 2021-10-23

**Authors:** Jessica Chitwood-Brown, Gary E. Vallad, Tong Geon Lee, Samuel F. Hutton

**Affiliations:** 1 Horticultural Sciences Department, University of Florida, Gainesville, FL 32611, USA; jchitwood@ufl.edu (J.C.-B.); tonggeonlee@ufl.edu (T.G.L.); 2 Plant Pathology Department, University of Florida, Gainesville, FL 32611, USA; gvallad@ufl.edu; 3 Gulf Coast Research and Education Center, University of Florida, Wimauma, FL 33598, USA; 4 Plant Molecular and Cellular Biology Graduate Program, University of Florida, Gainesville, FL 32611, USA

**Keywords:** tomato, tomato wild relatives, breeding, genetics, linkage drag, gene pyramiding, durable resistance

## Abstract

For over a century, breeders have worked to develop tomato (*Solanum lycopersicum*) cultivars with resistance to Fusarium wilt (*Fol*) caused by the soilborne fungus *Fusarium oxysporum* f. sp. *lycopersici*. Host resistance is the most effective strategy for the management of this disease. For each of the three *Fol* races, resistance has been introgressed from wild tomato species, predominately in the form of R genes. The *I, I-2, I-3*, and *I-7* R genes have each been identified, as well as the corresponding Avr effectors in the fungus with the exception of *Avr7*. The mechanisms by which the R gene protein products recognize these effectors, however, has not been elucidated. Extensive genetic mapping, gene cloning, and genome sequencing efforts support the development of tightly-linked molecular markers, which greatly expedite tomato breeding and the development of elite, *Fol* resistant cultivars. These resources also provide important tools for pyramiding resistance genes and should support the durability of host resistance.

## 1. Introduction

Tomato (*S. lycopersicum*) is one of the most important vegetable crops in the world, with over 180 million tons produced worldwide in 2019 and just over 5 million ha harvested (Faostat, http://www.fao.org/faostat/en/#data/QC/visualize, accessed on 14 September 2021). Between 1999 and 2019, the global area harvested increased by 27% while production increased by 66% (Faostat, http://www.fao.org/faostat/en/#data/QC/visualize, accessed on 14 September 2021). This gain in productivity is the result of research in many areas, notably the improvement of tomato varieties through breeding efforts. 

Tomato breeding has focused on improving traits important to growers and consumers, including fruit quality characteristics, yield, and disease resistance. Because tomato is susceptible to more than 200 pests and pathogens [1,2], breeding for appropriate disease resistances is crucial to the success of a tomato cultivar. However, unless a production area is under high disease pressure, resistance alone is not likely to make a cultivar successful. Tomato growers must produce fruit that satisfy market demands and meet industry standards in order to make a profit, and under ordinary circumstances, a new cultivar with disease resistance must perform at least comparably to existing susceptible cultivars with respect to fruit quality traits, yield, and other critical characteristics. 

Cultivated tomato is considered to have low genetic diversity due to bottlenecks in domestication and the subsequent effects of artificial selection, but wild tomato species harbor a great deal of variation [3,4]. For over a century, tomato breeders have utilized the genetic diversity within wild tomato species to introduce desirable traits, especially disease resistance genes, by crossing these with cultivated tomato [4]. In this review, we illustrate the efforts of tomato breeding for resistance to Fusarium wilt, including how disease resistance was identified in wild tomato species, the cloning of resistance genes, and the transition from phenotypic selection to marker assisted breeding with currently available tools.

## 2. Fusarium Wilt Description

**Figure 1:**
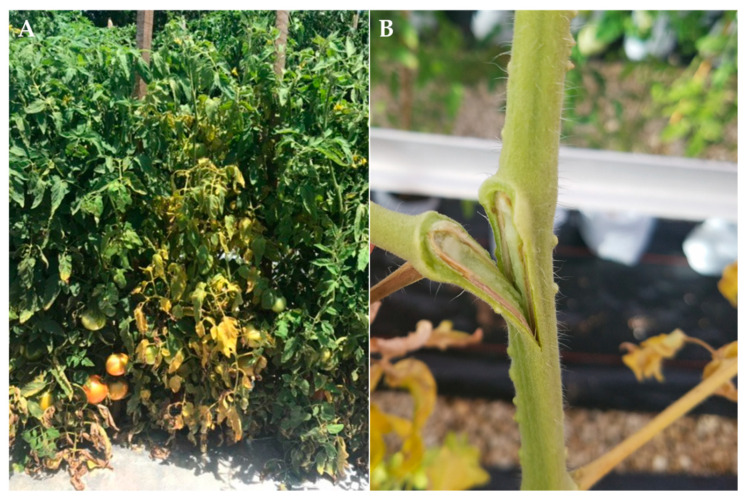
(**A**) Disease symptoms in a susceptible tomato (*S. lycopersicum*) plant grown to near maturity in field conditions. (**B**) Vascular browning in the stem of a susceptible tomato plant.

*F. oxysporum* (Schlecht. emend. Snyder & Hansen)*,* is a cosmopolitan soil fungus that exists as a saprophyte due to its ability to degrade lignin and other complex carbohydrates and is a common epiphyte and endophyte of plant roots [5,6]. However, within *F. oxysporum* exists more than 120 different specialized forms capable of causing severe disease losses on diverse vegetable, field, and plantation crops, although each form is limited to a range of hosts and designated as *formae specialis* (f. sp.) [7]. Fusarium wilt of tomato, caused by *F. oxysporum* f. sp. *lycopersici* (*Fol*), threatens US and global tomato production for both processing and fresh-market systems. *Fol* was first described in England in 1895 and has since been found in more than 40 countries [8]. The fungus penetrates plant roots before colonizing the vascular tissue. Initial disease symptoms begin as pronounced chlorosis and wilting of lower basal leaves that progresses acropetally to the upper leaves (Figure 1). These symptoms are often asymmetrical, restricted to one or two branches of the plant or even to one side of a leaf. As symptoms progress, wilting can occur either on the entire plant or one side, and early wilting may be more noticeable in the afternoon, with the plant appearing to recover overnight [8]. Browning in the vascular tissue of the stems as the pathogen colonizes the vascular tissue is commonly observed (Figure 1). Eventually, the disease leads to a rapid decline of the plant, accelerated fruit ripening, and plant death. Disease development is favored by sandy, acidic soils and warm temperatures (25–28 °C) [9,10]. 

*Fol* produces several asexual structures, including micro- and macroconidia and thick-walled chlamydospores that can survive in the soil for up to ten years [8]. Inoculum may be introduced into uninfested soil via the aerial dissemination of conida from the surfaces of infected plants under certain conditions [11]. The pathogen may also be spread on infected transplants or through the movement of contaminated soil on equipment, workers, or packing boxes [8]. Once introduced into a field, *Fol* is almost impossible to eliminate, as it can survive nearly indefinitely in soil as either chlamydospores in plant residues or as a common root epiphyte/saprophyte on numerous weeds without causing disease [12]. In the absence of control strategies, such as fumigation and host resistance (which is the focus of this review), *Fol* can result in up to complete crop loss [13]. 

## 3. *Fol* Control Strategies

Efforts to control *Fol* have included cultural, biological, chemical, and host resistance strategies to varying degrees of success. Altering the soil pH using lime and nitrogen amendments resulted in a reduction of disease incidence for all three races of *Fol* but did not eliminate crop losses [10]. Long fallow (where fields are not cultivated for a certain duration of time) or crop rotations are recommended to help reduce soil levels of *Fol* [8]. Jones et al. [14] observed reduced disease incidence and increased yields when a field was left uncultivated for a 9-month period. Corato et al. [15] reported a reduction in the total amount of spores and pathogenicity of *Fol* when tomato crops were rotated with durum wheat compared to tomato monoculture. While these strategies may reduce disease incidence, the benefits of crop rotation or the duration necessary to reduce *Fol* or similar soilborne pathogens to an acceptable level in tropical and subtropical production systems has not been adequately addressed. Further, similarly to many specialty crops in the U.S., the economics of tomato production do not allow for abandoning problematic fields or developing additional fields, and land rotations are limited due to the intense nature of tomato production. Thus, these strategies are not widely used in commercial tomato production. Studies have also investigated the utility of biological control [16,17,18]. However, these studies take place almost entirely in controlled greenhouse or growth chamber settings and further study into field efficacy of these control measures is needed. Nonetheless, like the previous control methods described, biological control measures do not eliminate the disease entirely and, to date, there are no reports of this strategy being implemented successfully on a commercial scale. 

In many places throughout the world, commercial tomato production requires soil fumigation to limit the impact of soilborne pathogens on production. From the 1970′s until 2014, the US tomato industry relied heavily on the preplant fumigant methyl bromide to maintain economical production levels [19,20,21]. Methyl bromide, a fumigant that quickly volatilizes once applied to soil, provided control against many soilborne pathogens in addition to nematodes and weeds over a broad range of environmental conditions, and had greater efficacy for controlling *Fol* than most cultural practices. However, designation as an ozone depleting chemical in 1993 under the Montreal Protocol led to the global phasing out of methyl bromide usage. Access to limited quantities of methyl bromide for tomato production through successive critical use exemptions ended 31 December 2014. 

Several registered alternative fumigants to methyl bromide exist (such as chloropicrin, dimethyl disulfide, 1,3-dichloropropene, and numerous isothiocyanate generators), although all lack the broad-spectrum activity and the volatility that made methyl bromide so highly effective [20]. Placement of the fumigant within the soil was demonstrated to affect soil levels of *Fol*, such that supplemental application of the chemicals alongside the raised soil bed reduced disease incidence [22,23]. However, this did not consistently result in an increase in yield [22]. The transition away from methyl bromide has been associated with losses of nearly $4000 per acre in Florida, one of the largest production areas for fresh-market tomatoes in the USA [21].

Host resistance is the most effective control strategy for *Fol*.

## 4. Breeding for Resistance

Efforts to develop tomato cultivars with resistance to *Fol* began in the 1900s at agricultural research stations across the USA, and some of the first tests were conducted in Florida in 1905 [24]. Resistance to the three races of *Fol* was discovered in wild tomato accessions, many of which have been used to study resistance and some to introduce resistance into cultivars for tomato production regions around the world [24,25,26]. Most of the genes identified for *Fol* resistance, and all the genes used commercially, are single, dominant resistance genes, or R genes.

Resistance to *Fol* was first identified by Bohn and Tucker [24] after extensive screening of wild tomato relatives in search of resistance which could be crossed with cultivated tomato. The researchers identified one resistant accession of *S. pimpinellifolium*, Missouri accession 160 (PI79532), which contained a single, dominant resistance locus they termed *I* for “Immunity”. This source was easily crossed into *S. lycopersicum*, and ‘Pan America’, containing the *I* gene, was later released as the first cultivar with *Fol* resistance [27]. The *I* gene was mapped to chromosome 11 [28].

A second race of *Fol* (*Fol2*) was subsequently reported by Alexander and Tucker [29], and resistance was again discovered in wild tomato germplasm [25]. However, efforts to breed for resistance were not commenced until the 1960s, when Florida’s tomato industry suffered tremendous crop losses due to outbreaks of *Fol2* [30]. PI126915, a naturally occurring hybrid between *S. pimpinellifolium* and *S. lycopersicum* [25], possessed simply-inherited, dominant *Fol2* resistance in addition to resistance to *Fol1* and other fungal pathogens including gray leaf spot (*Stemphylium* spp.) and Cladosporium (*Passalora fulva*) [30,31]. Cirulli and Alexander [32] determined the *Fol1* and *Fol2* resistances in this accession were conferred by two independent genes, and they designated the gene for *Fol2* resistance as *I-2*, which was also genetically mapped to chromosome 11 [33,34,35]. ‘Walter’ was released in 1969 as the first commercial variety with resistance to both *Fol1* and *Fol2* [36].

The third *Fol* race (*Fol3*) was initially reported in Australia in 1979 and then in Florida in 1982 [37,38]. Resistance to race 3 in the *S. pennellii* accession PI414773 was first described by McGrath et al. [39], and Scott and Jones [40] soon after identified a dominant *Fol3* resistance locus from the *S. pennellii* accession LA716. Both of these resistances were designated *I-3*, and the LA716-derived resistance developed by Scott and Jones [40] has served as the primary source of *Fol3* resistance for cultivar development around the world. Bournival et al. [41] mapped *I-3* to chromosome 7, linked with the isozyme marker *Got-2.* Interestingly, the resistance introgressed from PI414773 was later determined to be based not on *I-3*, but on a separate locus on chromosome 8 that was designated *I-7* [42,43]. 

Resistance beyond that to *Fol3* has also been observed in PI414773 and LA716, although the loci responsible for this resistance have been contested. McGrath et al. [39] reported that two genes in PI414773 were responsible for resistance to *Fol2*, and one of these was determined to be allelic with the *I-2* gene. The location of the second gene, although not demonstrated at the time, likely corresponds to *I-7*, which was recently demonstrated to confer resistance to *Fol2* as well as to *Fol3*. LA716 is also resistant to races 1 and 2 [40], and several studies have implicated the involvement of *I-3* or of nearby genes in each of these responses. Based on phenotypes of recombinants developed by Bournival et al. [44], the possibility of a separate gene linked to *I-3* and conferring resistance to *Fol1* was supported, but cosegregation of *Fol2* with *Fol3* resistance led to the conclusion that *Fol2* resistance was conferred by *I-3* itself or a by a tightly linked gene. Sarfatti et al. [45] likewise investigated the relationship between race 1 resistance and the *I-3* locus, and these authors described a separate *I1* locus that mapped to a 37 cM region on chromosome 7 but is distinct from *I-3*. Based on disease responses of a panel of recombinant lines having various combinations of the *I*, *I-2*, and *I-3* genes, Scott et al. [33] also demonstrated an intermediate *Fol2* effect by *I-3* or by a tightly linked gene, and they further supported the alleged *I1* locus. Despite the commonalities among these studies, all were conducted during a time of limited marker coverage and prior to knowledge of the precise locations of each of the introgressed resistance genes. More recently, Do et al. [46] challenged the existence of *I1*, instead concluding that the *I-3* gene itself confers a level of resistance to races 1 and 2. Using recombinant lines and *Fol1* and *Fol2* isolates with knockouts of the Avr3 effector (which is necessary for *I-3-*mediated resistance), they observed that lines which lacked the *I-3* locus were susceptible to race 2 and that a functional Avr3 was necessary for intermediate resistance to races 1 and 2. Although the existence of *I1* or an unknown *Fol2* resistance gene linked to *I-3* is therefore unlikely, it may still be useful to challenge lines containing *I-3* alone or containing a knock-out of *I-3* with *Fol1* and *Fol2* to better understand the effect *of I-3* against races 1 and 2.

Three loci conferring partial resistance to *Fol2* were also identified by Sela-Buurlage et al. [47]. Using 53 introgression lines carrying various chromosomal segments from *S. pennellii* accession LA716 within a *S. lycopersicum* background [48], the authors identified the *S. pennellii I-5* and the *I-6* loci on chromosomes 2 and 10, respectively. In addition, the greater susceptibility of one line carrying a *S. pennelli* introgression on chromosome 2 between markers CT75 and TG91 prompted the conclusion that a *S. lycopersicum* resistance allele is present at this locus, which the authors named *I-4*. But besides being named, the effects of these loci have neither been validated in separate populations nor have they been deployed commercially. 

## 5. Gene Cloning

The division of *Fol* into three races on the basis of overcoming resistance genes implies a gene-for-gene model for the host-pathogen system between the tomato *I* genes and *Fol* effectors. By this model, each *I* gene encodes a protein product that confers resistance dependent on a specific effector produced by *Fol*. Several effectors have been identified as a series of proteins detected within tomato xylem following *Fol* infection and are encoded by Secreted in xylem (*SIX*) genes, some of which are also designated as avirulence or *Avr* genes. 

Prior to the release of a published tomato genome [49], only *I-2* had been cloned and characterized [34]. In recent years, however, *I*, *I-3*, and *I-7* have been identified (Table 1), providing knowledge of each of the commercialized *I* genes [34,42,50,51]. The corresponding effectors required for resistance conferred by *I*, *I-2*, and *I-3* have also been identified as *Avr1* (*SIX4*), *Avr2* (*SIX3*), and *Avr3* (*SIX1*), respectively (Table 1) [52,53,54].

Catanzariti et al. [51] identified *Solyc11g011180* on chromosome 11 as the *I* gene, which encodes an atypical membrane-anchored leucine-rich repeat (LRR) receptor-like protein (RLP). *I* has several features which contrast with other known plant defense genes encoding LRR-RLPs. Some of these features are as follows: it does not belong to a gene cluster, introns are contained in the coding sequence, and specificity for Avr1 is defined by the C-terminus of the protein [51]. 

*I-2* was the first of the *I* genes to be isolated, and resistance is dependent on the effectors *Avr2* (*SIX3*) and *SIX5* ([34,54,55]. The *I-2* gene, *Solyc11g071430*, is also on chromosome 11 and encodes a coiled-coil (CC) nucleotide-binding (NB) LRR protein [34]. *I-2* is part of the *I2C* gene family, a complex of genes with structural similarities to NB-LRR resistance genes [34,56]. Although Ori et al. [56] reported that one gene in the cluster besides *I-2* (*viz. I2C-1*) conferred partial *Fol2* resistance in transgenic plants, Simons et al. [34] contradicted this, instead demonstrating that resistance is only given by *I-2*. The latter group suggested the reason for this discrepancy lay in the use by Ori et al. [56] of a tomato background with the *I* gene. In contrast, Simons et al. [34] had used material with no other *Fol* resistance, and they further stated that disease symptoms are less severe in plants infected with race 2 when race 1 resistance is present, although there are no published data to support this claim. Houterman et al. [54] found that *SIX3* was necessary for *I-2* resistance and thus called it *Avr2.* However, Ma et al. [55] later found that an additional SIX gene, *SIX5*, was also required for *I-2* resistance, as a knock-out of either *SIX3* or *SIX5* eliminated resistance and both were required for full pathogenicity. *SIX3* and *SIX5* also share a promoter region that controls their co-expression, with *SIX5* interacting with Avr2 to facilitate cell-to-cell movement of Avr2 within the host via plasmodesmata [57,58]. Three separate point mutations within the *SIX3 (Avr2)* gene have been described to prevent recognition by *I-2* but do not affect pathogenicity, resulting in the emergence of race 3 [54]. 

The *I-3* gene was identified as *SpSRLK-5* or *Solyc07g055640* and encodes an S-receptor-like-kinase (SRLK) on chromosome 7 [50]. *I-3-*mediated resistance is dependent on *Avr3* (*SIX1*), the first avirulence factor of *F. oxysporum* identified [52]. Transgenic plants containing *SpSRLK-5* challenged with an *Fol3* knock-out mutant that lacked *Avr3* (*Fol3*Δ*Avr3*) were susceptible, confirming that *SpSRLK-5* is the *I-3* gene. Unlike most plant disease resistance genes, *I-3* as a member of an SRLK gene family does not include a LRR domain; *I-3* is only the second resistance gene known to encode an SRLK, the first being the *Pi-d2* gene from rice [50]. *SpSRLK-5* is located among several SRLK genes in the *I-3* region that vary in their protein sequence similarity with one another, but the function of these SRLK paralogs has not been investigated. Interestingly, *SpSRLK-6* shares 91% sequence identity with *SpSRLK-5*, and disease development in transgenic lines with the *SpSRLK-6* paralog was less severe than in susceptible controls [50]. Thus, it is possible that this gene is also involved in a separate disease resistance response. 

Although first discovered by McGrath et al. [39], *I-7* was not identified as a separate locus from *I-3* until the 2006 report of Lim et al. [43]. The *I-7* gene (*Solyc08g077740*) encodes an LRR-receptor-like-protein and is borne on a small (approximately 210 kb) introgression on chromosome 8, which was identified using an RNA-seq analysis of root transcripts after previous genome wide marker screens failed to locate the introgression [42,59]. The Avr effector protein(s) corresponding to *I-7*-mediated resistance have not yet been identified. *I-7* also confers resistance to *Fol1* and *Fol2* based on seedling disease assays in which *I-7* was overexpressed in the *Fol* susceptible ‘Moneymaker’ background via transformation, though the effect of *I-7* against *Fol1* and *Fol2* under native expression has not been demonstrated. 

*I-4*, *I-5*, and *I-6* have not been cloned, and the mechanisms of these resistances have not been explored.

## 6. Linkage-Drag Associated with *I-3* Resolved

Although cultivars containing *I-3* have been commercially available since the mid 1990’s, breeders have experienced considerable difficulty developing commercially acceptable *Fol3* resistant hybrids. Some of the earliest *I-3* parents had greater susceptibility to blossom-end rot [60], and smaller fruit size was also demonstrated in plants that were homozygous for *I-3* compared to plants heterozygous for the gene [61]. Recently, Chitwood–Brown et al. [62] found that the *I-3* introgression decreases fruit size by approximately 21%. Furthermore, Hutton et al. [63] demonstrated that the *I-3* introgression in advanced breeding material contributed as much as a 20% increase in the severity of bacterial spot symptoms caused by *Xanthomonas perforans* race T4 in *I-3/I-3* plants relative to *i-3/i-3* plants. 

Li et al. [64] developed recombinant inbred lines (RILs) which segregated for different portions of the *S. pennellii* introgression by screening for cross-over events near the *I-3* gene. Using field disease screens, they demonstrated that increased sensitivity to bacterial spot co-segregated with the proximal portion of the *S. pennellii* introgression, above the *I-3* gene, regardless of whether the *I-3* gene itself was present or absent. These findings collectively suggested that increased sensitivity to bacterial spot is not due to the *I-3* gene itself but results from linkage with negative alleles. Building on this work, Chitwood–Brown et al. [62] reduced the *I-3* introgression to approximately 140 Kb, encompassing only 13 annotated genes besides *I-3* and excluding the proximal region previously implicated. Field evaluations of segregating backcross populations demonstrated that the reduced *I-3* introgression is free of linkage with both bacterial spot sensitivity and reduced fruit size. Germplasm containing the reduced *I-3* introgression is available, providing breeders with improved *Fol3* resistance resources for use in cultivar development [62].

## 7. Gene Expression and Host-Pathogen Interactions

Although the exact mechanisms by which the *I* genes confer resistance are not well understood, many studies have explored gene expression to attempt to elucidate the host–pathogen interactions. Early investigations utilized grafting between ‘Pan America’ (containing *I*) and ‘Bonny Best’ (*Fol* susceptible) and found that ‘Pan America’ rootstocks were sufficient for providing resistance to ‘Bonny Best’ scions while the reciprocal combination resulted in susceptibility. Thus, they concluded that resistance was localized to the roots [65]. However, Snyder et al. [66] demonstrated in ‘Pan America’ that although the fungus traveled beyond the roots and upward into the xylem of the stem, the plants did not always show foliar symptoms. They found no evidence of an inhibitory substance produced by the plant in the xylem and further reported that resistance functions in living cells throughout the plant, not only in the roots.

Nonetheless, the first point of contact between the pathogen and the host plant occurs in the roots, and many studies have examined expression of resistance genes in the roots, in addition to searching for interactions between the gene transcripts and fungal effectors in this tissue. Gonzales–Cendales et al. [42] in fact identified the *I-7* gene using RNA-seq to detect transcripts in root tissue that corresponded with resistance. In contrast, *I-3* is expressed at greater levels in leaf tissue than in the roots, where it is barely detectable [50]. Mes et al. [67] studied expression of the *I-2* gene using GUS reporting (β-glucuronidase reporter gene fused to *I-2* promotor) and detected expression in roots, stems, and leaves with the highest levels of expression in xylem parenchyma cells. Interestingly, *I-2* is expressed regardless of infection by *Fol2* [67]. They also demonstrated that containment of fungal colonization in resistant plants takes place in xylem parenchyma cells, indicating a possible site for *Avr2*/*I-2* interaction, although direct interaction between the two proteins was not observed. Houterman et al. [54] also studied the relationship between *I-2* and *Avr2* and demonstrated using agroinfiltration in *Nicotiana benthamiana* that Avr2 can be recognized intracellularly. However, direct interaction between the proteins has yet to be observed. 

Other attempts to study protein-protein interactions have likewise proved unsuccessful. Coexpression of *I* and *Avr1* in tobacco induced necrosis, which was helpful for confirming the identity of the *I* gene, but a direct interaction was not found [51]. Catanzariti et al. [50] utilized yeast two hybrid assays to investigate *I-3* and *Avr3* using various domains of *I-3*, but they were also unable to detect any interaction. Furthermore, coexpression of these two genes in tobacco did not result in any detectable phenotype. 

All tested *Fol* isolates carry *SIX1* (*Avr3*) and *SIX2* [52,68]. However, Rep et al. [52] observed that a collection of race 1 isolates were virulent on a tomato line containing *I-3*, even though they contained an intact *Avr3,* and the authors suggested these isolates overcame *I-3*-mediated resistance by some other fungal factor. Upon identification of *Avr1* (*SIX4*), Houterman et al. [53] reported that in addition to acting as an avirulence factor in the presence of the *I* gene, this effector suppressed resistance conferred by the *I-2* and *I-3* genes. Therefore, based on the current model of the *Fol*-tomato pathosystem, neither *I-2* nor *I-3* should provide effective resistance to race 1 strains, even though *Fol1* contains *Avr2* and *Avr3.* Consistent with this, Do et al. [46] reported that *I-3* conferred only partial resistance to *Fol1* due to suppression by Avr1, although the level of resistance was not clearly defined. Tomato breeders have therefore been advised to continue selecting for at least the *I* and *I-3* genes for cultivar development. Interestingly, Gonzalez–Cendales et al. [42] reported that the *I-7* gene confers resistance to *Fol1* and *Fol2* and is not suppressed by Avr1, a possible indication that this gene functions in a resistance pathway independent of the *I-3* gene. 

Although the function of *SIX* genes as effectors has been described, their presence alone is not necessarily indicative of pathogenicity. Jelinski et al. [69] identified *F. oxysporum* isolates that were recovered from the soils of tomato fields with a history of Fusarium wilt, which possessed the full repertoire of *SIX* genes but were nonpathogenic on tomato. It is possible that other chaperone genes, similar to *SIX5*, may be required for pathogenicity and host resistance [55]. Furthermore, those *SIX* genes required for pathogenicity in *Fol* lie within an accessory chromosome (also referred to as a supernumerary, B, or lineage-specific chromosome) that can be horizontally transferred between isolates, conferring pathogenicity to otherwise nonpathogenic isolates [70,71]. Combined with the lack of any observation of direct interaction between protein products of the *I* genes and *Avr* genes, addressing this gap in the understanding of the *Fol*-tomato system would be helpful for breeders to make more informed decisions as to which *I* genes are best to deploy and how they should be pyramided.

## 8. Phenotypic Selection Methodologies

Phenotypic screening has always been an important aspect in the advancement of disease resistance. Although such screens were formerly an indispensable component of all selection efforts, modern breeding programs often utilize molecular markers to guide their selections. Even so, phenotypic screening and selection remains necessary for identifying resistance sources, for gene discovery efforts, and for validating resistance in advanced materials. 

**Figure 2:**
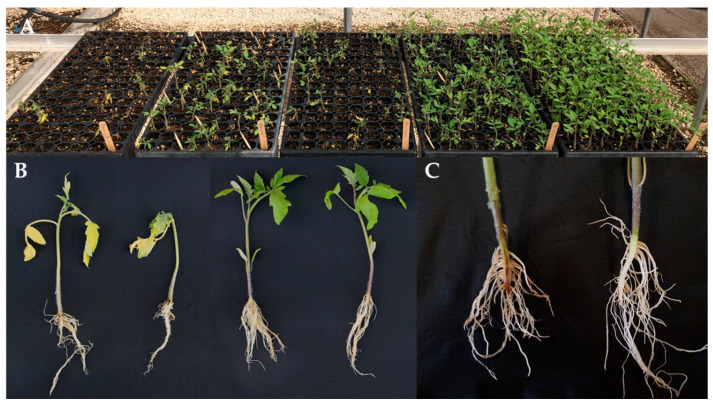
Symptoms observed in *Fol3* seedling disease assays. (**A**) From left to right, trays contain ‘Bonny Best’ (susceptible to all three races), ‘Manapal’ (contains *I*), ‘Horizon’ (contains *I* and *I-2*), ‘Tristar’ (contains *I*, *I-2*, and *I-7*), and Fla. 7946 (contains *I*, *I-2*, and *I-3*). (**B**) Two susceptible plants on the left are compared with two resistant plants on the right. (**C**) Root dissection reveals the vascular browning in a susceptible plant (left) compared with a resistant plant (right).

The earliest tests to identify *Fol* resistance were conducted in fields that were naturally infested with the pathogen [24]. Soil could also be artificially infested by adding cultures of the fungus grown in oats or cornmeal. Field testing, however, was cumbersome and was limited by the amount of time, land, and resources required to test at most a few thousand plants per season [72]. Field testing was also hampered by the presence of diseases other than Fusarium wilt and by differences in environmental conditions among regions [72]. Variation in the naturally occurring inoculum load of the soil further confounded results, often producing “escapes”–or genetically susceptible plants that remain healthy in the screen. Artificial inoculation of soil, both in the field and the greenhouse, was used to reduce the number of escapes and improve breeding efforts [24]. After experimenting with numerous inoculation methods, Wellman [72] introduced a technique in which the fungus was directly applied to seedling roots by immersing roots in a suspension of hyphae and spores. Seedlings were grown in a greenhouse until approximately four true leaves had developed, and then roots were washed free of soil and dipped in the inoculum. The seedlings were then transplanted into steam-sterilized soil and maintained in a greenhouse at 25 to 30 °C, an optimal temperature range for disease development. This technique, which became known as the Wellman root-dip method, was well-suited for quickly screening large numbers of plants and obtaining consistent, repeatable results. Although some field testing continued, the Wellman root-dip method served as the basis of Fusarium wilt disease assays from that point forward, and this technique, or modifications to it, are most frequently described in the literature. Common modifications include adjustments to the inoculum concentrations (which typically range between 1 × 10^6^, and 1 × 10^7^ conidia/mL) or to seedling age at the time of the inoculation (which commonly ranges between cotyledon and fourth true leaf state) [33,42,52,62]. Seedlings are typically evaluated after two to three weeks and are considered diseased if the plants are dead or exhibit clear stunting, wilting, chlorosis, enlarged stems, or stem collapse [33,42,62]. Plant roots may be dissected to determine if vascular browning is present, which is also indicative of *Fol* infection [33,62]. Figure 2 depicts symptoms commonly observed in seedling disease assays.

Although the Wellman root-dip method has several advantages over field testing, it is not without weakness. Wellman [72] noted that some seedlings appeared infected when the evaluation was done one week after inoculation, and that these plants seemed to outgrow the symptoms if given more time, later appearing normal in development. Thus, the timing of evaluation may contribute to the possibility that the disease response of a plant is falsely recorded during a seedling assay. Inoculum concentration and the age of the plant at the time of infection has also been reported to influence resistance response [73,74]. Some lines can appear to be susceptible in root-dip disease assays but have much less disease incidence when subjected to field tests [74]. As an example, Chitwood–Brown et al. [62] observed that resistance conferred by *I-7* was significantly impacted by inoculum concentration, where the proportion of healthy plants decreased from 86% to 23% as the inoculum concentration increased from 10^5^ to 10^7^ spores per mL, respectively. Escapes may also occur using the Wellman root-dip method of screening. Alexander and Hoover [25] pointed out the significance that this type of assay places on escapes and recommended increasing the number of tests to compensate in order to select resistant plants for breeding purposes. Another consideration includes the bias towards dominant, major resistance genes this screening method is likely to have.

Phenotypic disease screens remain a critical component of breeding for resistance to *Fol*, particularly in gene discovery studies. However, the limitations of seedling disease assays using the Wellman root-dip method must not be ignored. Although plants which survive the screen are likely to contain alleles which confer a level of resistance, other potentially useful resistance factors that would contribute to effective field resistance may be missed. 

## 9. Marker-Assisted Breeding

The introduction of genetic markers has revolutionized plant breeding. This has been especially important in breeding for disease resistances, as breeders have often been able to reduce their dependence on costly disease assays or on natural disease pressures, and instead utilize routine marker assays to track the presence of resistance genes in their breeding populations. 

The study of linkage mapping in plants opened the door to the ability to utilize genetic markers in plant breeding. Several types of molecular markers exist, and the degree to which they are used often depends on the technology required. Isozyme markers were among the first developed and utilized. Tanksley and Rick [75] developed a linkage map of isozyme markers in tomato and suggested their usefulness for introgressing genes from wild tomato species. Bournival et al. [41] identified tight linkage between the isozyme marker *Got-2* and *I-3*, the first marker for this gene. Restriction fragment length polymorphisms (RFLPs) allowed an improved degree of specificity because of the saturation of these markers across the tomato genome. *I-2* was found to be linked to RFLP marker TG105 [76], and Tanksley et al. [77] reported that *I-3* was linked to RFLP markers TG128, TG217, and TG170. 

As sequencing technologies improved and tomato genome sequences were made available, DNA sequence variations, such as simple sequence repeats (SSRs), insertion/deletions (indels), and single nucleotide polymorphisms (SNPs) were exploited for marker development. Cleaved amplified polymorphic sequence (CAPS) markers, which make use of SNPs and include restriction enzyme digestion, have been used extensively for tomato breeding applications and genetic studies, including the discovery of the *I, I-3*, and *I-7* genes [59]. Sequence characterized amplified region (SCAR) markers can be developed using indels and cost less than CAPS markers because they do not require digestion by restriction enzymes [59]. However, indels do not occur as frequently across the genome as SNPs and may, therefore, be less useful in certain research situations such as gene mapping.

To facilitate marker assisted selection, we have compiled a list of sequence polymorphisms for *I*, *I-2*, and *I-3*, providing a resource for marker development in a single location (Supplementary Table S1). This is especially helpful for *I* and *I-2*, which are absent in only a few of the genome sequences currently available to the public. For more information describing the breeding lines and methodology used, see Supplementary Methods. 

**Figure 3:**
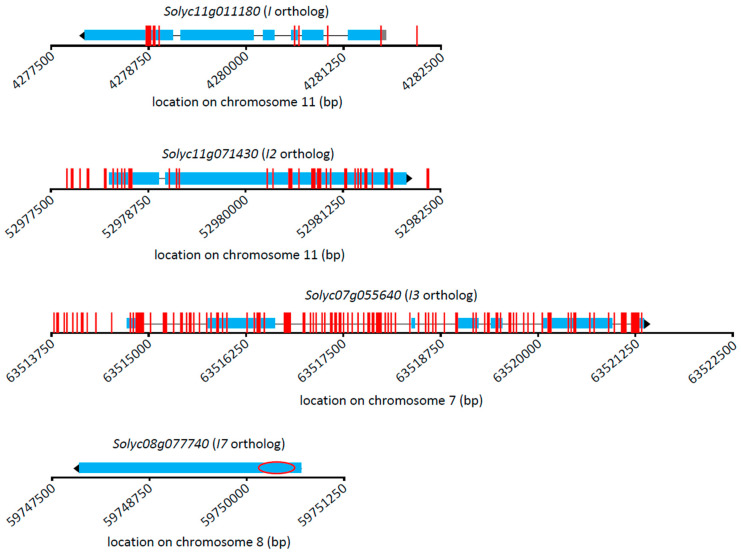
Single nucleotide polymorphism (SNP) sites across *I* gene orthologs in tomato (*S. lycopersicum*). Vertical red lines show locations of SNP sites. Ortholog gene models are shown at correct *x* axis positions for Heinz 1706 (*S. lycopersicum)* reference (blue bar, exons; black line, introns; and gray bar, untranslated region). Ortholog gene names are given above the gene model. The red circle in *Solyc08g077740* shows the location of a cleaved amplified polymorphic sequence marker previously designed for *I7*. A table showing SNP information for *I*, *I-2*, and *I-3* can be found in Supplementary Table S1.

Figure 3 displays polymorphisms detected in the orthologs and flanking regions of each gene, and detailed information for each polymorphism can be found in Supplementary Table S1. The lowest number of SNPs for any gene was 11, which were identified for the *I* ortholog by comparing Fla. 7907, Fla. 7946, Fla. 8653, and Fla. 8916 to the *Fol* susceptible cultivar ‘Yellow Pear’. Fifty-one SNPs were identified for the *I-2* ortholog by comparing the same four Fla. breeding lines to Heinz1706. The *I-3* ortholog contained the greatest number of SNPs, 148, identified by comparing Fla. 7907 and Fla. 7946 (*Fol3* susceptible) with Fla. 8653 and Fla. 8916 (*Fol3* resistant). These results will aid in the development of markers which are more tightly linked with, or located within, their respective genes. 

## 10. Durability of Resistance

All *Fol* resistance genes currently used in commercial tomato cultivars are R genes. The durability of R genes is typically dependent on the ability of the pathogen to mutate in order to overcome resistance, a well-known limitation to use of R gene-mediated resistance in plant crops around the world [78]. It is thought that mutations in the *SIX* genes of *Fol* over time have led to the breakdown of resistance and the emergence of new races, following a zig-zag model of plant-pathogen interaction [79]. However, there is evidence suggesting that the tomato-*Fol* relationship is more complex than this model or the gene-for-gene model implies. *Fol2* was first reported just four years after the release of ‘Pan America’, the first cultivar with resistance to *Fol* [29]. It is known that *Fol2* lacks *Avr1*, which is associated with increased aggressiveness and required for *I*-mediated resistance [52,54]. While it was undoubtably the selection pressure imposed via the use of the *I* gene which led to *Fol2* becoming the dominant race impacting tomato production, it is possible that this variation among isolates existed prior to the deployment of the *I* gene. Furthermore, the dependence of *I-2*-mediated resistance on two fungal effectors, the suppressive function of *Avr1*, and the conspicuous absence of detectable interactions between any of the *I* genes and fungal effectors challenge the oversimplified interpretation of a gene-for-gene model in this system.

Although the efficacy of *I* was lost relatively quickly after its deployment, the *I-2* gene provided resistance for nearly 20 years until the emergence of *Fol3*, which occurred in separate locations: Australia in 1979, in Florida in 1982, and later in California in 1988 [37,38,80]. Houterman et al. [54] identified three independent point mutations in *Avr2* which lead to the emergence of race 3 in isolated *Fol* populations, and it is likely that race 3 originated from local race 2 populations, rather than by introduction such as that on contaminated transplants [80]. Elias and Schneider [81] theorized that genetic determinants responsible for the development of new races may already exist in the pathogen population. Cultivars containing *I-3* have been successfully used for the control of *Fol3* over more than 30 years, but this resistance has not been as ubiquitously deployed as the *I* or *I-2* genes, and therefore, selection pressure for mutation in the pathogen has not been as intense. How much time remains before the pathogen overcomes *I-3*-mediated resistance?

Resistance based on quantitative trait loci (QTL) is considered a more durable and, thus, in some cases, the more preferred type of resistance compared to R genes [82]. Quantitative resistance is controlled by multiple genes, each contributing a small effect to the resistance phenotype, rather than resistance being conferred by a single gene. Mutation in a single *Avr* gene within the pathogen is unlikely to overcome such resistance, and pathogens that do overcome QTL resistance gain only a marginal advantage [78]. However, breeding for QTL-based resistance can prove challenging, especially for tomato, in which commercial cultivars are hybrids and disease resistance is often introgressed from wild tomato species. Traits introgressed from wild tomato are typically associated with linkage drag effects. Thus, developing commercial cultivars with resistance based on multiple introgressions can therefore be quite challenging, especially when effective R genes are readily available. It may, however, be possible to replicate the durability of QTL-based resistance by pyramiding R genes, especially those representing different modes of action between the resistance genes and the effectors they recognize [83,84]. Pyramiding R genes is an established approach in the development of disease resistant cultivars and stacking multiple genes for resistance to *Fol3* would likely produce durably resistant tomato cultivars. 

Already, *I*, *I-2*, and *I-3* are commonly stacked in commercial cultivars. The *I-3* and *I-7* genes represent different classes of R genes that may have distinct modes of action [42]. *I-3* and *I-7* were each introgressed from *S. pennellii* [39,40], and this species may harbor additional *Fol* resistance genes. The identification and introgression of novel *Fol3* resistance alleles could be especially useful for pyramiding with *I-3* and *I-7* to promote the durability of *Fol3* resistance. 

## 11. Conclusions and Future Outlook

Disease resistance remains the most effective strategy for management of Fusarium wilt, and the work to discover, utilize, and understand resistance for each of the three races as they emerged has spanned a century. In every case, resistance has been introgressed from wild tomato species in the form of major, dominant R genes. However, the manner in which these genes function to provide protection from *Fol*, and specifically how they recognize and interact with the corresponding *Fol* effectors, remains unknown. Although there have been efforts to explore the expression of the *I* genes and elucidate gene function, interpretation of the findings has not clear. Both *I-2* and *I-3* were detected in root, stem, and leaf tissues [50,67]; but whereas expression of *I-2* was detected at the highest level in the xylem parenchyma cells where infection by *Fol2* was also halted [67], *I-3* was almost undetectable in roots [50]. Further study is necessary to shed light on the interactions between the tomato host and *Fol*.

Although breeding for resistance to most diseases in tomato has primarily focused on the use of R genes, exploring quantitative resistance may be vital to the durability of resistance. Wild species likely harbor additional *Fol* resistance alleles, some of which may be nonrace specific major genes or QTL-based resistances. With the genetic resources available today, mining for these resistance loci in the sequences of wild accessions and incorporating them into commercial varieties is much less cumbersome than in the past. 

Although the pursuit of QTLs from wild species could prove challenging given potential for linkage-drag effects, there is opportunity to pursue QTLs affecting resistance that exist in cultivated backgrounds. ‘M82′, which lacks the *I-2* gene, is reported to be partially resistant to *Fol2* [47,85], and Sela-Buurlage et al. [47] reported a *S. lycopersicum Fol2* resistance locus from ‘M82′ and located on chromosome 2. Similarly, ‘Marglobe’, which lacks *I*, was reported to segregate for levels of resistance to *Fol1* [85]. Chitwood-Brown et al. [62] also demonstrated significantly lower *Fol3* disease incidence in Fla. 8059 relative to other susceptible controls, all of which lacked *I-3*. While the underlying mechanisms responsible for these observations are unknown, there are many points of host-pathogen interaction which lead to either infection and colonization by the fungus or recognition and resistance by the host. Roots present many barriers to infection, including root exudates, the structure of tissues, and the timing and magnitude of response to invasive growth of a pathogen [86,87,88]. Finally, Gao et al. [89] reported that symptom expression was determined by the ability of a plant to restrict the colonization of the fungus in the vascular tissue. Given the variation that exists among cultivars, it is likely that at least some of these traits are heritable and may be combined to increase the effectiveness of disease resistance. 

Taken together, such approaches have the potential to greatly increase the efficacy and durability of resistance, thereby contributing vital tools for tomato growers to utilize in their management of this important disease. 

## Figures and Tables

**Table 1:** Resistance genes introgressed from wild tomato species and their corresponding effectors of *Fusarium oxysporum* f. sp. *lycopersici*.

R Gene	Gene Class	Locus ID	Source	Effector Recognized
*I*	LRR-RLP	*Solyc11g011180*	*S. pimpinellifolium*	Avr1 (Six4)
*I-2*	CC-NB-LRR-RLP	*Solyc11g071430*	*S. pimpinellifolium*	Avr2 (Six3)
*I-3*	SRLK	*Solyc07g055640*	*S. pennellii*	Avr3 (Six1)
*I-7*	LRR-RLP	*Solyc08g077740*	*S. pennellii*	unknown

## Data Availability

Data is contained within the article or Supplementary Material.

## Supplementary Materials

The following are available online at https://www.mdpi.com/article/10.3390/genes12111673/s1, Single nucleotide polymorphisms (SNPs) in the Solanum lycopersicum ortholog of each Fusarium wilt resistance gene were predicted from aligned whole-genome sequencing (WGS) read data to the SL4.0 version of the Heinz 1706 tomato genome assembly [90]. Illumina whole-genome raw read data sets used in this study were as follows: one i germplasm accession [Yellow Pear, Sol Genomics Network (https://solgenomics.net), susceptible to all three races of Fol], one i-2 accession (Heinz 1706, susceptible to Fol2 and Fol3), two i-3 (Fla. 8653, Fla. 8916, susceptible to Fol3), and two I-3 (Fla. 7907, Fla. 7936, resistant to all three races of Fol). All Fla. breeding lines possess both I and I-2 and are large-fruited, fresh-market breeding lines with determinate growth habit. ‘Yellow Pear’ is small fruited, indeterminate, unimproved (heirloom) cultivar. Further, intracultivar SNPs in Heinz 1706 were identified in two individual Heinz 1706 plants [LA4345; seed was obtained from the Tomato Genetics Resource Center (https://tgrc.ucdavis.edu)] and excluded in this study. BWA-MEM algorithm [version 0.7.17 [91]] with paired-end options was used to align the reads to the reference genome assembly [90]. After removing SNP calls supported by fewer than six reads in the WGS data, we identified SNPs in each gene.
