# Supplementary material for: Breeding for Resistance to Fusarium Wilt of Tomato: A Review
Source: Genes (Basel). 2021 Oct 23;12(11):1673. doi: 10.3390/genes12111673 (PMC8624629; doi:10.3390/genes12111673)
Supplement: Supplementary file 1 [file genes-12-01673-s001.zip › genes-1424419-Supplementary/Supplementary Methods.pdf]

Supplementary Methods

Single nucleotide polymorphisms (SNPs) in the *Solanum lycopersicum* ortholog of each Fusarium wilt resistance gene were predicted from aligned whole-genome sequencing (WGS) read data to the SL4.0 version of the Heinz 1706 tomato genome assembly [91]. Illumina whole-genome raw read data sets used in this study were as follows: one *i* germplasm accession [Yellow Pear, Sol Genomics Network (https://solgenomics.net), susceptible to all three races of *Fol*], one *i-2* accession (Heinz 1706, susceptible to *Fol2* and *Fol3*), two *i-3* (Fla. 8653, Fla. 8916, susceptible to *Fol3*), and two *I-3* (Fla. 7907, Fla. 7936, resistant to all three races of *Fol*). All Fla. breeding lines possess both *I* and *I-2* and are large-fruited, fresh-market breeding lines with determinate growth habit. 'Yellow Pear' is small fruited, indeterminate, unimproved (heirloom) cultivar. Further, intracultivar SNPs in Heinz 1706 were identified in two individual Heinz 1706 plants [LA4345; seed was obtained from the Tomato Genetics Resource Center (https://tgrc.ucdavis.edu)] and excluded in this study. BWA-MEM algorithm [version 0.7.17 [92]] with paired-end options was used to align the reads to the reference genome assembly [91]. After removing SNP calls supported by fewer than six reads in the WGS data, we identified SNPs in each gene.
